# Evaluation of real-time RT-PCR for the quantification of FCoV shedding in the faeces of domestic cats

**DOI:** 10.1016/j.jfms.2007.10.010

**Published:** 2008-04

**Authors:** Charlotte Dye, Christopher R. Helps, Stuart G. Siddell

**Affiliations:** Small Animal Hospital, Department of Clinical Veterinary Science, University of Bristol, Langford House, Langford, Bristol BS40 5DU, UK

## Abstract

Faecal samples were taken from cats living in multi-cat households with endemic feline coronavirus (FCoV) infection. Total RNA was extracted from faecal suspensions and FCoV RNA was quantified using a real-time reverse transcriptase-polymerase chain reaction (RT-PCR) assay. The real-time RT-PCR threshold cycle (*C*_T_) values were consistently high suggesting that the samples contained very little viral RNA. However, experiments in which RNA extracted from FCoV-infected cell culture supernatants was combined with RNA extracted from faecal suspensions revealed the presence of faecal factors that significantly inhibited the reverse transcription reaction. Consequently, three methods of RNA extraction were investigated and RNA dilution was undertaken to investigate whether the effects of the faecal inhibitors could be reduced. Our results show that using the QIAgen RNA mini kit for RNA extraction and dilution of the RNA samples helps to reduce the inhibitory effects. However, because the extent of the inhibitory effects varied between faecal samples, accurate quantification proved difficult. We, therefore, conclude that although real-time RT-PCR provides an excellent method for detecting the presence of viral shedding, quantification of FCoV RNA in faecal material has to take into account the possible effects of RT-PCR inhibitors. It is, therefore, essential that all new assays, and the methods of sample preparation, are carefully evaluated before being used in a clinical setting.

Feline coronavirus (FCoV) infection is extremely common in cats and in the United Kingdom approximately 40% of the domestic cat population have positive serum antibody titres (Pedersen 1995). Where cats are housed together in multi-cat households the prevalence is even higher at around 90% (Addie and Jarrett, 1992, Sparkes et al., 1992, Addie, 2000). Natural infections with FCoV are usually transient, although a significant percentage of infections may become persistent (Addie and Jarrett 2001). Most infections are asymptomatic or result in mild, self-limiting gastrointestinal disease. However, in a small percentage of animals, a fatal multi-systemic, immune-mediated disease known as feline infectious peritonitis (FIP) occurs (Pedersen 1995). The devastating effects of FIP have made FCoV infection a significant concern for veterinarians, cat owners and breeders alike.

FCoVs are highly infectious. They are spread predominantly by the faeco-oral route but also in oro-nasal secretions and in urine (Hoskins 1993). Cats recovering from coronavirus infection will shed virus in their faeces and are a potential risk to other susceptible cats. Most cats will shed virus for a period of a few weeks to months either continuously or transiently (Addie and Jarrett, 2001, Lutz et al., 2002b). Occasionally persistent carriers are found which will shed virus indefinitely (Addie and Jarrett 2001). In multi-cat households a large proportion of cats (75–100%) will be shedding coronavirus at any given time (Pedersen 1995). Each individual cat will undergo cycles of infection, shedding, recovery and re-infection (Foley et al 1997a). Close contact between infected and susceptible cats facilitates the most effective transmission and is important in the recirculation of virus allowing maintenance of endemic infection. However, coronavirus can persist in the environment for 3–7 weeks (Hoskins 1993) and the risk of transmission via fomites also exists. There is no known vertical transmission of the virus.

At the present time there are no effective treatments available for cats with FIP and the disease is invariably fatal. This means that, currently, controls to prevent FCoV infection are the most effective method for controlling FIP disease. A number of control strategies have been used based on either elimination or minimisation of infection. Environmental controls are the most widely used tool and good hygiene techniques are essential. Coronaviruses are inactivated by most household detergents (Maris 1990), but appropriate cattery design is required to facilitate effective disinfection and this is a major drawback in many ‘household breeding’ situations. Keeping small group sizes (not more than five cats housed together), reducing levels of intercurrent disease and minimising stress levels will also reduce the incidence of FIP (Addie and Jarrett 1992). Isolation and early weaning protocols may also be beneficial to reduce the exposure of young kittens that are at high risk of infection. For multi-cat households with endemic FCoV infection separation of cats within the household according to their level of faecal shedding has been advocated to help to accelerate the rate of viral load reduction (Foley et al., 1997a, Addie and Jarrett, 2001, Lutz et al., 2002b). This strategy requires frequent monitoring of FCoV in faecal samples. Viral particles remain stable in faeces for at least 10 days and faecal shedding can be assessed using reverse transcriptase-polymerase chain reaction (RT-PCR) assays to detect the viral genome (Lutz et al 2002b). Recently, quantitative real-time RT-PCR methods have become available enabling the amount, as well as the frequency, of virus shedding to be assessed (Gut et al 1999). This could potentially be a useful tool as high-level shedders pose a greater threat of disease transmission (Foley et al 1997b). Using this approach, a recent study showed that the majority of cats (78%) were ‘low-level’ shedders with shedding frequencies of less than 30%. Approximately 20% of the cats were termed medium-level shedders with shedding frequencies of 30–90% and only 1% of the cats were high-level shedders with shedding frequencies exceeding 90% (Lutz et al 2002a). Taking four faecal samples over a 3-week period has been suggested to give a good indication of viral load (Lutz et al 2002b).

The presence of faecal inhibitors that interfere with the function of the reverse transcriptase and DNA polymerase enzymes makes faecal RT-PCR assays notoriously difficult (Chui et al., 2004, Radstrom et al., 2004). The aim of this study was, therefore, to investigate the significance and occurrence of inhibitors that might interfere with real-time RT-PCR assays for the detection of FCoV RNA in faecal samples from domestic cats.

## Materials and methods

### Maintenance of cell lines

Crandell Reese feline kidney cells (CrFK) were a kind gift from Dr Allison German of the University of Bristol. The cells were maintained in Dulbecco's modified Eagle's media (DMEM) with 10% (v/v) foetal bovine serum (FBS), 100 units/ml penicillin G sulphate, 100 μg/ml streptomycin sulphate and 1× non-essential amino acids. Cell monolayers were propagated in 75 cm^2^ tissue culture flasks kept at 37°C in 5% CO_2_ humidified incubators.

### Preparation of virus stocks

Feline infectious peritonitis virus (FIPV), strain 79-1146 was purchased from the American Type Culture Collection (VR-2202, ATCC). Virus stocks were prepared by infecting subconfluent (∼80%) CrFK cell monolayers at a high multiplicity of infection. Cell monolayers were incubated with virus diluted in media for 45 min at 37°C in 5% CO_2_ humidified incubators. The cell culture supernatant was then discarded and replaced with media. Cell culture supernatant containing virus was harvested at 8 h after infection and centrifuged at 800 × *g* for 5 min. An aliquot of the supernatant was taken for estimation of the virus titre by 50% tissue culture infectivity dose (TCID_50_) and the remainder was aliquoted and stored at −80°C.

### Faecal samples

Faecal material was obtained from 19 healthy cats living in multi-cat households with endemic FCoV infection. The samples were taken by the cat owners who removed fresh faecal pellets from the litter trays, placed them in sterile pots and posted them to the laboratory. Samples were stored for a maximum of 72 h at 4°C pending postage and 10% (w/v) faecal suspensions in phosphate buffered saline (PBS) were made immediately on receipt. The faecal suspensions were stored at −80°C.

### RNA extraction using the QIAamp viral RNA mini kit

RNA was extracted from cell culture supernatant and from 10% faecal suspensions using a QIAamp viral RNA mini kit according to the manufacturer's instructions. Briefly, 140 μl aliquots of samples were lysed under highly denaturing conditions to inactivate RNAses. Buffering conditions were then adjusted with ethanol to provide optimal binding of RNA to a silica-gel based capture membrane. Contaminants were washed away using two different wash buffers. The RNA was eluted in 60 μl of RNAse-free, low-salt buffer at room temperature and was stored at −80°C.

### RNA extraction using QIAamp DNA stool mini kit

RNA was extracted from 200 μl of a 10% faecal suspension in PBS using the QIAamp DNA stool mini kit according to the manufacturer's instructions. Briefly, the faecal samples were suspended in buffer ASL (QIAgen, UK), which is designed to remove inhibitory substances from stool samples. ‘InhibitEX’ was added to adsorb these substances, which were then removed by centrifugation. Following proteinase K treatment the samples were bound to a silica-gel based capture membrane, washed and then eluted in a low-salt buffer. The eluted RNA was stored at −80°C.

### Boom method for RNA extraction from faeces

A 20 μl aliquot of extraction matrix (size fractionated silica) was added to 1 ml of L6 buffer (8.3 M guanidinium isothiocyanate, 83 mM Tris–HCl pH 6.4, 36 mM ethylene diamine tetra-acetic acid (EDTA), 2% (v/v) Triton-X-100) containing 200 μl of a 10% faecal suspension. After vortexing for 10 s the samples were incubated for 15 min at room temperature. Following centrifugation at 16,000 × *g* for 15 s the RNA pellet was washed twice with 1 ml of L2 buffer (8.3 M guanidinium isothiocyanate, 83 mM Tris–HCl pH 6.4), twice with 1 ml of 70% ethanol and once with 1 ml of acetone. The RNA was dried at 56°C for 5 min and was re-suspended in 50 μl of RNAse-free water containing 15 units of human placental ribonuclease inhibitor (HPRI). Following a 15-min incubation at 56°C the suspension was centrifuged at 16,000 × *g* for 2 min and the RNA containing supernatant was stored at −80°C.

### Real-time RT-PCR assay design

Oligonucleotide primers for reverse transcription (1b) and PCR (P009 and P010) and a *Taqman* probe (P9/10P) were designed using the ‘Primer3’ software package (Table 1). The choice of primers was based upon a consensus sequence derived from published sequence data for five FCoV isolates obtained from the Genbank database (A22378, AB086904, AB086903, AB086902 and X56496). A 171 nucleotide region spanning the membrane-nucleocapsid gene junction was chosen for amplification because these genes represent the most abundant mRNA's and nucleotide sequence in this region of the genome is well conserved (Schreiber et al., 1989, Jouvenne et al., 1990, Tobler et al., 1993).

**Table 1:** RT-PCR oligonucleotide primers and oligonucleotide probe

Oligonucleotide name	Use	Nucleotide sequence	Position in FIPV 79-1146 genome∗
P1b	RT primer	TCATAGCGGATCTTTAAACTTCTC	29012–29035
P009	Forward PCR primer	AGCAACTACTGCCACRGGAT	26655–26674
P010	Reverse PCR primer	GGAAGGTTCATCTCCCCAGT	26807–26826
P9/10P	*Taqman* probe	AATGGCCACACAGGGACAACGC	26781–26802

∗ Genbank accession number DQ010921.

### Real-time RT-PCR reaction

Superscript II RNAse H^−^ reverse transcriptase was used to reverse transcribe viral RNA. RNA samples containing 5 pmol of primer 1b were incubated at 65°C for 5 min and then chilled on ice. The RNA-primer mix was then added to a 20 μl reaction containing 15 units of human placental ribonuclease inhibitor (HPRI), 1 mM dNTP, 0.01 M dithiothreitol (DTT) and 1× first strand buffer (Invitrogen, UK). The reaction was incubated at 42°C for 2 min before the addition of 200 units of Superscript II RT enzyme. The reaction was incubated at 42°C for 50 min followed by 94°C for 2 min. Samples were immediately chilled on ice and stored at −20°C. Real-time PCR reactions were done in duplicate using HotStarTaq mastermix according to the manufacturer's instructions. A 25 μl reaction containing 2 μl of cDNA template (10% of the RT reaction volume), 1× PCR mix (supplied by manufacturer), 0.25 μM primer P009, 0.25 μM primer P010 and 1.5 mM MgCl_2_ was made with 0.2 μM of the 5′FAM/3′BHQ-1 labelled P9/10P *Taqman* probe. The reaction was incubated at 95°C for 15 min. The cDNA was then amplified using 45 cycles of 95°C for 10 s, 56°C for 15 s and 72°C for 15 s.

## Results

### Real-time RT-PCR assay optimisation

Viral RNA from the supernatants of CrFK cells infected with the laboratory stain FIPV 79-1146 was used as template for optimisation of the real-time RT-PCR assay. Temperature gradients revealed an optimum annealing temperature of 59°C and 10-fold template dilution series revealed good reaction efficiency (95.9%). Melt curves (d*F*/d*T* vs temperature) done using an SYBR green I reporter system were used to confirm the absence of non-specific products (data not shown). Subsequently, ‘quantification graphs’ (fluorescence vs cycle number) were used to compare threshold cycle (*C*_T_) values using a standard fluorescence threshold of 100 rfu. Confirmation of reaction efficiencies for use of the assay with field strain virus was not possible due to low *C*_T_ values precluding the use of serial dilutions. However, any reduction in the RT-PCR reaction efficiency using field strain RNA template would most likely result from nucleotide discrepancies within the PCR primer annealing sites. The P009, P010 and *Taqman* probe annealing sites on the RNA from several of the clinical FCoV strains were sequenced to check for primer mismatches. This revealed good sequence conservation with a maximum of two nucleotide mismatches per oligonucleotide (data not shown). Further investigation revealed that small reductions in RT-PCR reaction efficiency resulting from these primer mismatches could be eliminated by reducing the PCR annealing temperature from 59 to 56°C.

### FCoV RNA in faecal samples

CrFK cells were infected with the laboratory strain virus FIPV 79-1146 and RNA was extracted from the cell culture supernatant. Following reverse transcription the cDNA was diluted 1:1000 with AE buffer (QIAgen, UK) and 5 μl was used as template in the real-time PCR assay. Faecal material obtained from cats living in multi-cat households was used to make a 1:10 faecal suspension in PBS and RNA was extracted using a QIAgen viral RNA mini kit. The faecal RNA was reverse transcribed and the cDNA was diluted to a 50 μl volume with AE buffer. Five microlitres of the cDNA was used as template in the real-time PCR assay. Negative (water) controls were also included for both the RT and PCR steps. The positive control FIPV 79-1146 sample gave a low *C*_T_ value as expected and the negative controls failed to give any signal after 45 PCR cycles. All 13 faecal samples tested had very high *C*_T_ values (Table 2) suggesting that the concentration of viral RNA in the samples was extremely low. Significant degradation of the RNA was felt to be unlikely as FCoV RNA has been shown to remain stable within faecal samples for at least 10 days at room temperature (Lutz et al 2002b) and processing methods within the laboratory were specifically chosen to minimise this problem. Further investigations were, therefore, undertaken to investigate whether faecal inhibitors were a confounding factor.

**Table 2:** Real-time RT-PCR results for RNA samples extracted from the faeces of cats in multi-cat households with endemic FCoV infection

Sample	*C*_T_ value
FIPV 79-1146	20.4
Faeces 1	37.2
Faeces 2	36.3
Faeces 3	38.4
Faeces 4	36.0
Faeces 5	39.0
Faeces 6	(−)
Faeces 7	35.9
Faeces 8	37.4
Faeces 9	36.0
Faeces 10	36.5
Faeces 11	31.6
Faeces 12	33.2
Faeces 13	33.2
Water control	(−)

Results are expressed as threshold cycle (*C*_T_) values and positive (FIPV 79-1146) and negative (water) control samples are included. Negative results are represented by (−).

### Investigation of RNA extraction method

Three different methods of RNA extraction were used to investigate whether the effects of faecal inhibitors could be minimised during the RNA extraction process. Two QIAgen methods, the QIAamp viral RNA mini kit and the QIAamp DNA stool mini kit were used according to the manufacturer's instructions. A third method described by Boom et al (van der Hoek et al 1995) was also used. Each of the three methods was also used to extract RNA from FIPV 79-1146 infected cell culture supernatant and from uninfected media as positive and negative controls, respectively. For each sample the P009/P010 RT-PCR assay was run and water controls for the RT and PCR steps were also included. The QIAgen viral RNA mini kit achieved the lowest *C*_T_ values in the majority of the samples with the QIAgen stool DNA mini kit giving *C*_T_ values ranging from one to three cycles higher (Table 3). *C*_T_ values obtained using the Boom extraction method were inconsistent when compared to the QIAgen methods. The FIPV 79-1146 sample gave a *C*_T_ value 16 cycles higher than that achieved with the QIAgen viral RNA mini kit, yet in faecal sample 14 it gave almost the same *C*_T_ value as the QIAgen viral RNA mini kit. In the remaining faecal samples the Boom method gave *C*_T_ values ranging from two to six cycles higher than the Qiagen methods. Based on these results the QIAgen viral RNA mini kit was chosen as the method of faecal RNA extraction for subsequent work.

**Table 3:** Real-time RT-PCR results for RNA isolated from faecal samples using different methods of RNA extraction

	FIPV 79-1146	Faeces 14	Faeces 15	Faeces 16	Faeces 17	Media control	RNA water control	PCR water control
Boom extraction method	36.4	34.9	40.5	34.1	36.2	(−)	(−)	(−)
QIAgen viral RNA mini kit	20.2	35.0	34.5	31.8	32.8	(−)	(−)	(−)
QIAgen stool DNA mini kit	21.1	38.1	37.4	33.3	34.0	(−)	(−)	(−)

Results are expressed as threshold cycle (*C*_T_) values and positive (FIPV 79-1146) and negative (media and water) controls are included. Negative results are represented as (−).

### Investigation of faecal inhibitors

RNA was extracted from 140 μl of FIPV 79-1146 infected cell culture supernatant, from a 1:10 faecal suspension (faeces 19) and from uninfected media. Also, 100 μl of an identical FIPV 79-1146 infected cell culture supernatant was ‘spiked’ with 40 μl of either uninfected media or with the same faecal suspension and RNA was extracted using the same methods. Reverse transcription was undertaken using primer 1b and cDNA was amplified in the P009/P010 real-time PCR reaction. A water control sample was included and all samples were run in duplicate (Table 4). The FIPV 79-1146 samples spiked with faecal suspension gave *C*_T_ values ∼9 cycles higher than their media spiked equivalents. This indicates that the presence of faecal material prior to RNA extraction had decreased the apparent viral content of the original sample by a factor of ∼500 (2^9^).

**Table 4:** Comparison of threshold cycle (*C*_T_) values from the RT-PCR of FIPV 79-1146 infected cell culture supernatant ‘spiked’ with either faecal suspension or with uninfected media

	FIPV 79-1146	Faeces 19	Media	FIPV + faeces	FIPV + media	Water
*C*_T_ value run 1	24.9	37.9	(−)	38.8	28.4	(−)
*C*_T_ value run 2	24.9	37.7	(−)	39.0	28.4	(−)

Reactions were done in duplicate and the results of run 1 and run 2 are shown. Negative results are represented as (−).

### Inhibition of the reverse transcription reaction

RNA was extracted from FIPV 79-1146 infected cell culture supernatant and from a 1:10 faecal suspension (faeces 19). Three 20 μl reverse transcription reactions were done, each containing 10 μl of template as follows: tube 1: 3 μl FIPV 79-1146 RNA + 7 μl water, tube 2: 3 μl FIPV 79-1146 RNA + 7 μl faecal RNA, and tube 3: 3 μl water + 7 μl faecal RNA. The reactions were diluted to a volume of 50 μl with AE buffer (50 mM sodium acetate pH 5.3, 10 mM EDTA) and 5 μl of each was used as template in a real-time PCR reaction. Sample 1, containing viral RNA alone, had a *C*_T_ value ∼9 cycles lower than that of sample 2, containing viral RNA spiked with faecal suspension (Table 5). This indicates that the presence of RNA extracted from faecal suspension prior to reverse transcription had reduced the apparent viral concentration in the initial sample by a factor of ∼500 (2^9^).

**Table 5:** Inhibition of reverse transcription by faecal inhibitors

	FIPV RNA alone	FIPV RNA + faecal RNA	Faecal RNA alone	Water control
*C*_T_ value for run 1	17.5	26.8	38.8	(−)
*C*_T_ value for run 2	17.0	26.8	38.0	(−)

The results are expressed as threshold cycle (*C*_T_) values and duplicate results are shown as run 1 and run 2. Negative results are represented as (−).

### Inhibition of the PCR reaction

RNA was extracted from FIPV 79-1146 infected cell culture supernatant and from a 1:10 faecal suspension (faeces 19). Following reverse transcription with primer 1b the cDNA was diluted to a volume of 50 μl with AE buffer. Four PCR reactions were done each containing 5 μl of cDNA template as follows: tube 1: 2.5 μl FIPV + 2.5 μl water, tube 2: 2.5 μl FIPV cDNA + 2.5 μl faecal cDNA, tube 3: 2.5 μl faecal cDNA + 2.5 μl water, and tube 4: 5 μl water. Tubes 1 and 2, containing FIPV 79-1146 cDNA alone and FIPV 79-1146 cDNA spiked with faecal cDNA, respectively, both had the same *C*_T_ values (Table 6). This shows that the presence of cDNA derived from faecal suspension did not result in inhibition of the PCR reaction.

**Table 6:** Inhibition of the PCR reaction

	FIPV cDNA alone	FIPV cDNA + faecal cDNA	Water control
*C*_T_ value for run 1	17.8	18.2	(−)
*C*_T_ value for run 2	18.1	18.4	(−)

The results are expressed as threshold cycle (*C*_T_) values and duplicate results are shown as run 1 and run 2. Negative results are represented as (−).

### RNA dilution

RNA was extracted from FIPV 79-1146 infected cell culture supernatant. Also, 100 μl of the same FIPV 79-1146 infected cell culture supernatant was ‘spiked’ with 40 μl of faecal suspension (faeces 19) and RNA was extracted using the same method and solutions. Doubling dilutions of the ‘spiked’ RNA were made in RNAse-free water and 10 μl of each dilution was used as template in the P009/P010 real-time RT-PCR reaction. Positive (FIPV 79-1146 RNA) and negative (water) controls were included and all reactions were done in duplicate. The results are shown in Table 7. Instead of the expected increase in *C*_T_ values with increasing viral RNA dilution, the threshold cycle decreased with the first two dilutions (1:2 and 1:4). This suggests that the dilution of faecal inhibitors was having a greater effect than dilution of the viral RNA. At the 1:8 and 1:16 dilutions the *C*_T_ values rose steadily suggesting that further dilution was of no benefit. A 7-cycle improvement in the *C*_T_ value was seen at the 1:4 dilution which equates to an increase in the apparent initial virus concentration by a factor of ∼130 (2^7^). Subsequent testing revealed that the optimal RNA dilution factor was different for each individual faecal sample, making a standardised protocol for accurate quantification difficult to implement (data not shown).

**Table 7:** RNA template dilution

	FIPV 79-1146	FIPV + faeces	FIPV + faeces	FIPV + faeces	FIPV + faeces	FIPV + faeces	Water control
			1:2 dilution	1:4 dilution	1:8 dilution	1:16 dilution	
*C*_T_ value run 1	21.1	36.7	35.0	29.1	30.0	30.5	(−)
*C*_T_ value run 2	21.1	36.8	35.5	29.1	29.6	30.5	(−)

The results are expressed as threshold cycle (*C*_T_) values and duplicate results are shown as run 1 and run 2. Negative results are represented as (−).

## Discussion

The results of our study show that the quantification of FCoV RNA from faecal samples of domestic cats using real-time RT-PCR is problematic because of the presence of enzyme inhibitors. A series of ‘spiking’ experiments showed that this inhibition was a significant problem only for the reverse transcription reaction and not for the PCR amplification step. This finding could reflect the relative amounts of faecal-derived material in the two reactions (50% of the RT reaction volume are derived from faecal RNA compared with only 2% of the PCR reaction) or it may reflect the specific inhibition of an enzyme activity. The effect of these inhibitors was minimised by the use of the QIAgen viral RNA mini kit for RNA extraction and by dilution of RNA samples prior to reverse transcription. A 1:4 RNA dilution could be used empirically to ensure that false negative RT-PCR results were minimised. However, as our preliminary results have shown that faecal samples contained variable inhibitor concentrations, we believe that quantitative assessment of faecal viral RNA content using this method may often be inaccurate. Given the profound effects of faecal inhibitors found in this study it may be that some cats designated as low level shedders using similar assays could actually be shedding virus at much higher levels. Such results could have significant clinical relevance if used as part of screening programmes within households attempting to reduce viral loads.

The results of this study are in broad agreement with those of many other authors who have experienced problems amplifying nucleic acid from faecal material. For example, a recent study evaluating eight methods for extracting severe acute respiratory syndrome coronavirus (SARS CoV) RNA from faecal material concluded that the majority retained inhibitory substances preventing optimal amplification (Petrich et al 2006). However, several previously published studies quantifying FCoV faecal shedding using real-time RT-PCR have not reported significant problems associated with faecal inhibitors. It is possible that this problem has not been specifically addressed previously and that faecal shedding of FCoV may, therefore, have been commonly underestimated. However, it is also possible that alternative protocols may succeed in satisfactorily overcoming the problem. For example, in this study we only investigated the use of one reverse transcriptase enzyme (Superscript II RNAse H^−^) and it is possible that other RT enzymes may be less sensitive to the effects of inhibitors. Nevertheless, our results suggest that internal positive RT-PCR controls should be included in future assays and that the potential for variable levels of inhibitory factors in faecal samples should be kept in mind. Performing multiple assays using various RNA dilutions may also be of benefit. The conclusion is, therefore, not that accurate quantification of FCoV RNA in faeces is impossible but that each individual assay should be carefully evaluated before it is used in a clinical setting.

## References

[bib1] Addie D.D. (2000). Clustering of feline coronaviruses in multi-cat households. Veterinary Journal.

[bib2] Addie D.D., Jarrett O. (1992). A study of naturally occurring feline coronavirus infections in kittens. Veterinary Record.

[bib3] Addie D.D., Jarrett O. (2001). Use of a reverse-transcriptase polymerase chain reaction for monitoring the shedding of feline coronavirus by healthy cats. Veterinary Record.

[bib4] Chui L.W., King R., Lu P., Manninen K., Sim J. (2004). Evaluation of four DNA extraction methods for the detection of *Mycobacterium avium* subsp. paratuberculosis by polymerase chain reaction. Diagnostic Microbiology and Infectious Disease.

[bib5] Foley J.E., Poland A., Carlson J., Pedersen N.C. (1997). Patterns of feline coronavirus infection and fecal shedding from cats in multiple-cat environments. Journal of the American Veterinary Medical Association.

[bib6] Foley J.E., Poland A., Carlson J., Pedersen N.C. (1997). Risk factors for feline infectious peritonitis among cats in multiple-cat environments with endemic feline enteric coronavirus. Journal of the American Veterinary Medical Association.

[bib7] Gut M., Leutenegger C.M., Huder J.B., Pedersen N.C., Lutz H. (1999). One-tube fluorogenic reverse transcription-polymerase chain reaction for the quantitation of feline coronaviruses. Journal of Virology Methods.

[bib8] Hoskins J.D. (1993). Coronavirus infection in cats. Veterinary Clinics of North America Small Animal Practice.

[bib19] van der Hoek L., Boom R., Goudsmit J., Snijders F., Sol C.J. (1995). Isolation of human immunodeficiency virus type 1 (HIV-1) RNA from feces by a simple method and difference between HIV-1 subpopulations in feces and serum. Journal of Clinical Microbiology.

[bib9] Jouvenne P., Richardson C.D., Schreiber S.S., Lai M.M., Talbot P.J. (1990). Sequence analysis of the membrane protein gene of human coronavirus 229E. Virology.

[bib10] Lutz H, Gut M, Leutenegger CM, Schiller I, Wiseman A, Meli M (2002a) Kinetics of FCoV infections in kittens born in catteries if high risk for FIP under different rearing conditions. In: *Second International FCoV/FIP Symposium*. Glasgow, p. 20.

[bib11] Lutz H, Machler MR, Gut M, Leutengger C, Meli M (2002b). FCoV shedding pattern of privately owned cats under field conditions. In: *Second International FCoV/FIP Symposium*. Glasgow, p. 23.

[bib12] Maris P. (1990). Virucidal efficacy of eight disinfectants against pneumovirus, coronavirus and parvovirus. Annales de Recherches Vétérinaires.

[bib13] Pedersen N.C. (1995). An overview of feline enteric coronavirus and infectious peritonitis virus infections. Feline Practice.

[bib14] Petrich A., Mahony J., Chong S., Broukhanski G., Gharabaghi F., Johnson G., Louie L., Luinstra K., Willey B., Akhaven P., Chui L., Jamieson F., Louie M., Mazzulli T., Tellier R., Smieja M., Cai W., Chernesky M., Richardson S.E. (2006). Multicenter comparison of nucleic acid extraction methods for detection of severe acute respiratory syndrome coronavirus RNA in stool specimens. Journal of Clinical Microbiology.

[bib15] Radstrom P., Knutsson R., Wolffs P., Lovenklev M., Lofstrom C. (2004). Pre-PCR processing: strategies to generate PCR-compatible samples. Molecular Biotechnology.

[bib16] Schreiber S.S., Kamahora T., Lai M.M. (1989). Sequence analysis of the nucleocapsid protein gene of human coronavirus 229E. Virology.

[bib17] Sparkes A.H., Gruffydd-Jones T.J., Harbour D.A. (1992). Feline coronavirus antibodies in UK cats. Veterinary Record.

[bib18] Tobler K., Bridgen A., Ackermann M. (1993). Sequence analysis of the nucleocapsid protein gene of porcine epidemic diarrhoea virus. Advances in Experimental Medicine and Biology.

